# Combined life satisfaction of persons with stroke and their caregivers: associations with caregiver burden and the impact of stroke

**DOI:** 10.1186/1477-7525-9-1

**Published:** 2011-01-11

**Authors:** Aileen L Bergström, Gunilla Eriksson, Lena von Koch, Kerstin Tham

**Affiliations:** 1 Division of Occupational Therapy, Department of Neurobiology, Care Sciences and Society, Karolinska Institutet, Stockholm, Sweden; 2 Department of Occupational Therapy, University Hospital, Uppsala Akademiska Sjukhuset, Uppsala, Sweden; 3 Division of Neurology, Department of Clinical Neuroscience, Karolinska Institutet, Stockholm, Sweden; 4 Department of Occupational Therapy, Karolinska University Hospital, Stockholm, Sweden

## Abstract

**Background:**

Little is known about the life satisfaction of the person with stroke combined with their caregiver, i.e. the dyad, despite the fact that life satisfaction is an important rehabilitation outcome. The aim of this study was to describe the dyads combined life satisfaction and to understand this in relationship to the perceived impact of stroke in everyday life and caregiver burden.

**Methods:**

In this cross-sectional study, the life satisfaction of persons and their informal caregivers was measured in 81 dyads one year post stroke. Their global life satisfaction, measured with LiSat-11, was combined to a dyad score and the dyads were then categorized as satisfied, dissatisfied or discordant. The groups were compared and analyzed regarding levels of caregiver burden, measured with the Caregiver Burden scale, and the perceived impact of stroke in everyday life, measured with the Stroke Impact Scale (SIS).

**Results:**

The satisfied dyads comprised 40%, dissatisfied 26% and those that were discordant 34%. The satisfied dyads reported a significantly lower impact of the stroke in everyday life compared with the dyads that were not satisfied. As expected, dyads that were not satisfied reported a significantly greater caregiver burden compared with the satisfied dyads. The discordant group was further broken down into a group of dissatisfied and satisfied caregivers. The caregivers that were not satisfied in the discordant group perceived a significantly greater level of caregiver burden compared with the satisfied group. Even caregivers who were satisfied with life but whose care recipients were not satisfied reported caregiver burden.

**Conclusions:**

Measuring combined life satisfaction provides a unique focus and appears to be a feasible way of attaining the dyads' perspective. The findings suggest that those dyads with a discordant life satisfaction could be vulnerable because of the caregivers' reported caregiver burden. These findings support the importance of a dyadic perspective and add to the understanding of the reciprocal influences between the caregiver and recipient. This knowledge has clinical implications and contributes to the identification of possible vulnerable dyads in need of tailored support.

## Background

Persons with a stroke often perceive challenges and limitations in everyday life [1] and this in turn has been shown to be related to lower levels of perceived global life satisfaction [2-4]. The person with stroke often needs assistance from others [5], who take on the role of informal caregiver. Informal caregivers will often experience caregiver burden [6] and caregiver burden has been shown to be associated with a lower level of life satisfaction [7]. The reciprocal influences of the person with stroke and their caregiver have been shown in a number of different relations *e.g. *poor life satisfaction in persons with stroke has been shown to be related to burden in caregivers [8].

Reciprocal influences between the person with stroke and their caregiver have also been studied in a number of qualitative studies [9-11]. Moreover, caregiver role strain [12] and life satisfaction [13] have been shown to be influenced by mutuality, which includes the affective relationship involving shared activities in the couple [12]. One study showed that mutuality was the only variable that was a significant predictor of life satisfaction for persons with stroke, as well as their spouses in the long term [13], motivating a closer exploration of the dyad and their life satisfaction.

The importance of the informal caregivers' situation has been recognized by an amendment to Swedish law in 2009 (Social Services Act 2001, chapter 5, paragraph 10). This amendment emphasizes support in order to minimize the caregivers' physical and psychological strain, caregiver burden, and recognizes the caregivers' perspective in guiding interventions, such as support groups and home help [14]. This implies that within rehabilitation, the dyad could be viewed as one client, with the potential of benefitting from support. This view has also been suggested in recent research [15] but has not yet been introduced in stroke rehabilitation.

### Combined life satisfaction of the dyads

Life satisfaction is considered to be an important rehabilitation outcome [16] not only for the persons with stroke but for persons close to them as well, [17] such as caregivers. In this paper, life satisfaction is considered as the subjective view of how an individual perceives his or her life [18] and reflects the extent to which the person achieves his/her vital goals [17]. Although a number of studies have analyzed the life satisfaction of the person with stroke or their caregiver, little is known regarding the life satisfaction within the dyad. Life satisfaction of the person with stroke and their caregiver was combined and used in the present study. We defined combined life satisfaction as the perceived global life satisfaction [3] of two individuals in a dyad.

Two other studies have combined the life satisfaction of the person with stroke or brain injury with their spouse [19,20]. Both studies found similar results with approximately one third of the couples in agreement with being satisfied with their life as a whole, one third not in agreement, and one third of the couples were not satisfied. Eriksson and colleagues [20] also showed that the "joint" perceptions of life satisfaction were significantly related to couple's functioning in everyday life, and specifically to participation in leisure activities and social life. Despite the fact that two previous studies [19,20] have explored combined life satisfaction, the relationship between the dyads' combined life satisfaction and caregiver burden has not been established.

Considering the effects of a stroke on the everyday life for both persons and the reciprocal interactions between the two persons in the dyad, there is a need to illuminate how a stroke impacts both persons individually as well as together. This is in line with a number of studies calling for a combined patient- and caregiver-focused approach [21,22] and a need for research regarding the caregiver dyad [23]. A better understanding of the dyads situation could serve as a foundation for identifying vulnerable dyads in order to provide targeted support. The need for identifying vulnerable caregivers has been indicated [6] but research leading to the identification of vulnerable dyads is lacking. In order to gain a better understanding of the situation for the persons with stroke together with their caregivers, the focus of this study will be on the dyads' combined global life satisfaction and how it is associated with caregiver burden and the impact of stroke.

Thus, the aim of this study was threefold. Firstly, the aim was to describe the combined life satisfaction of two individuals making up the dyad. Secondly, the aim was to investigate the association of the combined life satisfaction with the persons with stroke perceived impact of the stroke in everyday life one year after onset. Lastly, the aim was to investigate the association of the combined life satisfaction with the caregivers' perceived level of caregiver burden one year after the stroke. Because of the known relationships between life satisfaction and caregiver burden [22] we hypothesized that the dyads' combined life satisfaction will be associated with the level of caregiver burden as well as to the perceived impact of stroke in everyday life.

## Methods

### Participants and procedures

The participants in the present study were recruited from a larger, hospital based study. All persons with a stroke diagnosis admitted to the stroke units within the Karolinska University Hospital, Stockholm, Sweden during the period of one calendar year were considered potential candidates for the hospital based study. After exclusions for persons declining participation, medical or ethical reasons, or incorrect diagnosis, a total of 349 persons with stroke were included in the larger study. The participants in the present study were recruited from those 349 persons.

All study participants were informed orally and in writing 3 to 5 days post stroke as to the overall plan and purpose for the research project, confidentiality, and the right to terminate the study. The person with stroke was asked to identify an acquaintance that would be considered his or her main, informal caregiver. This person could potentially be a spouse, family member or other acquaintance, living together with the person or not. They were then asked permission to contact the potential caregiver in order to receive information concerning the study. If the person with stroke did not identify a caregiver or give permission to contact the potential caregiver, they were not included in the present study.

Baseline data was extracted from the patients' medical records and the remaining data collection was via direct contact with the study participants at the rehabilitation clinic or in their home. All data was collected by specially trained research assistants (clinically experienced occupational and physical therapists). For the persons with stroke, data was collected at onset (i.e. at inclusion in the study) and at 12 months. Information regarding ADL was collected with the Barthel Index (BI) and the scores at inclusion were used to determine stroke severity [24]. Information regarding aphasia was collected at inclusion with the speech item of the Scandinavian Stroke Scale [25], which is a determination of the persons' ability in four increments (i. no aphasia ii. limited vocabulary or incoherent speech, iii. more than yes/no, but not longer sentences and iiii. only yes/no or less). For the caregivers, socio-demographic data was collected at 3 months post stroke during a visit to the person's home or if the caregiver was not available, via a questionnaire which was left to the caregiver and was to be returned by mail in a stamped envelope. The remaining instruments were administered approximately 12 months post stroke in the same manner.

This project was approved by the regional ethics committee, Stockholm, Sweden.

### Instruments

The following instruments were used one year post stroke, to measure the perceived life satisfaction of the dyads, the perceived level of impact of the stroke in everyday life for the persons with stroke and the perceived level of burden of care for the caregiver.

#### Life Satisfaction Checklist (LiSat-11)

The LiSat-11 [18] encompasses eleven items assessing overall and domain-specific life satisfaction. The first question in the checklist concerns global life satisfaction where the participants rate their satisfaction with life as a whole. The validity of using the global question as a measure of life satisfaction has been confirmed [3]. The responses to the global life satisfaction question were used in determining the combined life satisfaction of the dyad. The checklist uses a six-step ordinal self-rating scale ranging from (6) "very satisfying" to (1) "very dissatisfying". The results of each individuals response to the first question was dichotomized, with the scores of 5-6 meaning "satisfied", and the scores 1-4 meaning "dissatisfied". This is considered to be a valid scale reduction[18] and has been used in stroke studies [20,26,27]. To determine combined life satisfaction, the dichotomized results of the first question concerning global life satisfaction of the two individuals in the dyad were combined and then classified into 3 groups: satisfied, discordant (i.e. not in agreement) and dissatisfied. This was done according to a previous study [20]. The LiSat-11 has been used in studies of stroke samples [4,7,19,26] and has been found to be reliable over time for patients post stroke [3].

#### Stroke Impact Scale: (SIS)

The Stroke Impact Scale 2.0 [28] aims at measuring the perceived impact of stroke in everyday life through evaluation of different relevant domains for persons with stroke. The SIS is made up of 64 items in eight different domains: strength, hand function, mobility, activities of daily living (ADL) and instrumental ADL, emotion, communication, memory, and social participation. The greater the score (0-100), less impact is perceived on impairment, disability, health or quality of life. The SIS is a stroke specific outcome measure and was developed from the perspectives of persons with mild to moderate stroke [28]. The SIS has shown to be reliable, valid and sensitive to change [28] and has been frequently used [29-31]. A proxy version of the SIS [32] was used for those participants, unable to respond due to aphasia.

#### Caregiver Burden scale (CBs)

The Caregiver Burden scale [33] assesses the subjective burden of the persons assisting the person with stroke. The scale comprises 22 items, dealing with the caregiver's health, feeling of psychological well-being, relations, social network, physical workload, and environmental aspects. The questions are scored from 1 to 4 (not at all, seldom, sometimes, often). Scores range from 22 indicating no burden up to 88 indicating a great burden. The scale, which was based on a Swedish population, was developed for caregivers to patients with stroke and dementia [34]. The scale has been shown to have good construct validity and test-retest stability [33,34] and has been used in studies with caregivers to persons with stroke [22,35].

### Statistical analysis

Nonparametric statistics were chosen because the data were either nominal or ordinal level, the relatively small sample size, and because the variables were not normally distributed across the sample.

The Kruskal-Wallis (ANOVA by Ranks) was used first to determine if there was a difference between the groups of combined life satisfaction. The Mann Whitney U test was used for pair wise comparisons between the groups of combined life satisfaction regarding the caregiver burden, and the perceived impact of stroke in 8 different domains.

A *p *> .05 was considered non-significant (NS). In order to reduce the possibility of type I statistical errors, admittedly thereby running the risk of increasing the number of type II errors, the chosen level of significance for the multiple comparisons was set at *p *< 0.01.

Statistica (StatSoft Inc., version 8.0) was used for all statistical analysis.

## Results

Of the 349 persons with a stroke diagnosis in the original study, 54 (approximately 15%) were deceased one year post stroke, 45 persons had incomplete data, 76 declined or were lost to follow up and 93 had no identified caregiver. This left 81 persons with a caregiver (a total of 162 persons), at the one-year follow-up. The characteristics of the persons with stroke are shown according to the combined life satisfaction of the dyads in Table 1. Of the 81 participants, 69% had a mild stroke, 25% a moderate, 6% a severe stroke, their median age was 71 (range 32-92) and 67% were men.

**Table 1:** Characteristics of the persons with stroke according to the dyads combined life satisfaction

	Discordant dyads n = 28 (34%)	Dissatisfied dyads n = 21 (26%)	Satisfied dyads n = 32 (40%)	Total n = 81
Male/female *n *(%)	16 (57)/12 (43)	17 (81)/4 (19)	21 (66)/11 (34)	54 (67)/27 (33)
Median age in years *n *(range)	72.5 (49-92)	76 (53-88)	66 (32-84)	71 (32-92)
Civil status *n *(%)				
married/cohabitating	22 (78)	21 (100)	28 (87.5)	71 (88)
single	6 (22)	-	4 (12.5)	10 (12)
Children living at home *n *(%)	1 (4)	5 (24)	6 (19)	12 (15)
Born in Sweden yes/no *n *(%)	25 (89)/3 (11)	14(67)/7 (33)	25 (87)/7 (21)	64(79)/17(21)
Living conditions *n *(%)				
Home/apartment	28 (100)	20 (95)	32 (100)	80 (98.5)
Assisted living	-	1 (5)	-	1 (1.5)
Localization of stroke *n *(%)				
Left Hemisphere	8 (29)	7 (33)	13 (41)	28 (35)
Right Hemisphere	18 (64)	11 (52)	15 (47)	44 (54)
Unspecified	2 (7)	3 (14)	4 (12)	9 (11)
Type of injury *n *(%)				
Ischemic	24 (86)	18 (86)	28 (87.5)	70 (86)
Hemorrhage	4 (14)	3 (14)	3 (9)*	10 (12)
Stroke severity *n *(%)				
Mild	16 (57)	13 (62)	27 (84)	56 (69)
Moderate	9 (32)	7 (33)	4 (12)	20 (25)
Severe	3 (10)	1 (5)	1 (3)	5 (6)
Barthel Index median (QR) (at inclusion)	70 (62.5)	65 (55)	92.5 (20)	90.00 (55)
Barthel Index median (QR) (12 m. post stroke)	95 (17.5)	100 (20)	100 (0)	100 (10)
Aphasia *n *(%)				
No aphasia	21 (75)	13 (62)	24 (75)	58 (72)
Limited vocabulary/Incoherent speech	5 (18)	4 (19)	7 (22)	16 (20)
More than yes/no, but no longer sentences	-	3 (14)	1(3)	4 (5)
Only yes/no or less	1 (3.5)*	1 (5)	-	2 (2)
Global life satisfaction (12 m. post stroke) raw scores *n *(%)				
Score 1 (low)	-	1 (5)	-	1 (1)
Score 2 (low)	-	-	-	
Score 3 (low)	1 (3.5)	5 (24)	-	6 (7)
Score 4 (low)	8 (28.5)	15 (71)	-	23 (28)
Score 5 (high)	13 (46)	-	13 (41)	26 (32)
Score 6 (high)	6 (21)	-	19 (59)	25 (31)

*1 missing

**Figure 1:**
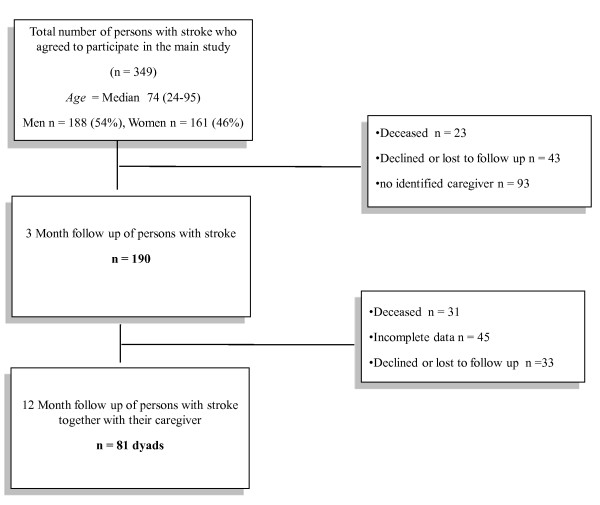
**Study Participants**.

Of the remaining 268 persons with stroke not included in the present study, 150 (56%) had a mild stroke, 53 (20%) had a moderate stroke and 65 (24%) had a severe stroke at inclusion. The median age for the 268 persons was 75 (range 24-95) and gender was equally distributed. A flow-chart of the participants with stroke is shown in figure 1.

The characteristics of the caregivers, of which 70% were women and 85% were partners to the person with stroke, are shown according to the combined life satisfaction of the dyads in Table 2.

**Table 2:** Characteristics of the caregivers (*n *= 81) according to the dyads combined life satisfaction.

	Discordant dyads n = 28 (34%)	Dissatisfied dyads n = 21 (26%)	Satisfied dyads n = 32 (40%)	Total caregiver information n = 81
Male/female *n *(%)	9 (32)/19 (68)	5 (24)/16 (76)	10 (31)/22 (69)	24 (30)/57 (70)
Median age in years (range)	65.5 (19-82)	73 (46-84)	63.5	66 (19-84)
missing *n *(%)	9 (32)	6 (28.5)	18 (56)	38 (47)
Caregiver *n *(%)				
partner	21 (75)	20 (95)	28(87.5)	69 (85)
son	1 (3.5)	-	1 (3)	2 (2)
daughter	3 (11)	-	1 (3)	4 (5)
other	1 (3.5)	-	-	1 (1)
missing	2 (7)	1 (5)	2 (6)	5 (6)
Living together with care recipient				
Yes/no	14 (50)/4 (18)	17 (81)/1 (5)	15 (47)/3 (9)	46 (57)/8 (10)
missing	10 (28)	3 (14)	14 (44)	27 (33)
Median Caregiver Burden Scale (QR)	41.5 (20.5)	52 (22)	27 (14.5)	39 (26)
Global life satisfaction raw scores *n *(%)				
Score 1 (low)	-	1 (5)	-	1 (1)
Score 2 (low)	1 (3)	1(5)	-	2 (2)
Score 3 (low)	2 (7)	6 (28)	-	8 (10)
Score 4 (low)	16 (57)	13 (62)	-	29 (36)
Score 5 (high)	7 (25)	-	24 (75)	31 (38)
Score 6 (high)	2 (7)	-	8 (25)	10 (12)

### Combined Life Satisfaction

The results shows that of the 81 dyads, 32 dyads (40%) were satisfied with life as a whole, while 21 (26%) were dissatisfied with life as a whole. The persons in 28 dyads (34%) responded differently, i.e. they had a discordant life satisfaction. In all, a total of 41 of the 81 caregivers were satisfied. This is shown in Table 3.

**Table 3:** Combined Life Satisfaction of the dyads

	*n *(%)	Male/female caregivers
Satisfied dyads	32 (40)	10/22
Discordant/satisfied caregiver	9	3/6
Discordant/dissatisfied caregiver	19	6/13
Dissatisfied dyads	21 (26)	5/16

### Combined life satisfaction and the impact of stroke

The results of the impact of stroke in everyday life and the association with the dyads combined life satisfaction are presented in Table 4. These results show that the persons with stroke in the dyads that were dissatisfied rated that their stroke had a greater impact on their life compared with those persons with stroke in the dyads that were satisfied. Furthermore, there were significant differences in the perceptions of the persons with stroke in the discordant dyads compared with the satisfied dyads regarding three aspects of physical functions of the SIS (strength, ADL and hand function) as well as participation and emotions. In contrast, the more cognitive aspects of the SIS, memory and communication, differed significantly between the discordant dyads and the dissatisfied dyads. Furthermore, the results of the SIS domain for communication showed that the median score was 92.85 indicating that the persons with stroke perceived a low impact on their communication ability.

**Table 4:** SIS domain scores and the relationships to the dyads' combined life satisfaction.

SIS-Scales	Total Median (IQ)	Median (IQ) Satisfied Dyads	Median (IQ) Dissatisfied Dyads	Median (IQ) Discordant Dyads	Discordant dyads compared with dissatisfied dyads (p value)	Discordant dyads compared with satisfied dyads (p value)	Dissatisfied dyads compared with satisfied dyads (p value)
Strength	75.00 (43.75)	100 (25)	68.75 (31.25)	62.5 (31.25)	N.S.	<0.01	<0.01
Memory	87.50 (21.87)	93.75 (12.5)	68.75 (31.25)	84.37 (21.87)	<0.01	N.S.	<0.01
Emotion	84.72 (27.77)	93.05 (9.72)	65.27 (18.05)	77.77 (23.61)	N.S.	<0.01.	<0.01
Communication	92.85 (16.07)	100 (7.14)	75 (26.78)	92.85 (14.28)	<0.01	N.S.	<0.01
ADL/IADL	87.50 (32.29)	100 (10.41)	70.83 (52)	81.25 (37.5)	N.S.	<0.01	<0.01
Mobility	87.50 (26.25)	96.25 (11.25)	77.5 (28.75)	78.75 (35)	N.S.	N.S.	<0.01
Hand function	90.00(40.00)	100 (22.99)	65.0 (45)	85 (50)	N.S.	<0.01	<0.01
Participation	80.55 (36.11)	100 (15.27)	61 (22.22)	75 (25)	N.S.	<0.01	<0.01

N.S. = not significant

### Combined life satisfaction and caregiver burden

**Figure 2:**
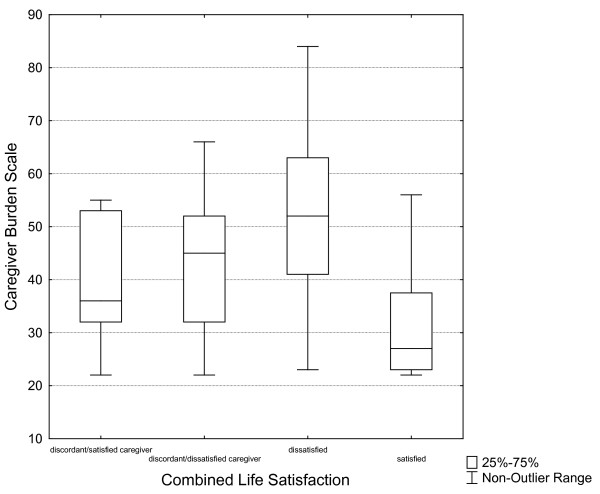
**Combined life satisfaction of the dyads in relation to the Caregiver Burden Scale**.

The associations between the dyads combined life satisfaction with caregiver burden are presented in figure 2. In order to get a better understanding of the dyads situation, we started by breaking down the discordant group. One group was identified where the person with stroke had a low life satisfaction while their caregiver had a high life satisfaction (n = 9 dyads) and the other group (n = 19 dyads) where the person with stroke had a high life satisfaction while their caregiver had a low life satisfaction. These four dyad groups of combined life satisfaction were then analyzed in relation to caregiver burden. The analysis showed that there was a significant difference in caregiver burden between the group of satisfied dyads and the dyads that were dissatisfied (*p *< 0.01). Also, there was a significant difference between the discordant group (with dissatisfied caregivers) and the caregivers in the satisfied group (*p *< 0.01) regarding caregiver burden.

There were no significant differences in the levels of caregiver burden between the two discordant groups. In other words, caregivers that were satisfied but their care recipients were not, reported caregiver burden. To further illustrate this, we refer the reader to Figure 2.

## Discussion

In order to ascertain more knowledge regarding the complex situation in everyday life of the person with stroke together with their caregiver, this study considered the two individuals as a dyad and combined their life satisfaction. By examining the association between the dyads' combined life satisfaction with the perceived impact of stroke on the one hand and the combined life satisfaction with caregiver burden on the other hand, this study attempted to achieve a dyadic perspective. The findings regarding the dyads combined life satisfaction, met our expectations as approximately 66% of the dyads had a congruent life satisfaction. Furthermore, the dyads combined life satisfaction was significantly associated with the perceived impact of stroke in everyday life and caregiver burden. In the dyads that were dissatisfied the impact of stroke and caregiver burden were significantly higher compared with those dyads that were satisfied with life as a whole.

The greater number of dissatisfied caregivers in the discordant group (19 of 28) was an unexpected result. The dissatisfied caregivers in the discordant group reported a significantly greater caregiver burden compared with caregivers in the satisfied dyads, indicating a potential vulnerability within the dyad and with the possibility of affecting the care recipient [1]. Even the caregivers who were satisfied with life but whose care recipients were not satisfied, expressed caregiver burden, suggesting other potentially vulnerable dyads. A possible interpretation of these results is that mutuality and reciprocal influences within the dyad, supported in previous studies [9-11], impacts everyday life after stroke and has clinical implications for identifying potentially vulnerable dyads.

The discordant dyads were divided in a study by Carlsson and colleagues [19] in the same way as in the present study but the opposite relation was found; only 9 percent of the spouses in the group of discordant dyads were dissatisfied. One can only speculate regarding the reasons for the differences in the studies. For example, hemisphere location of the insult may have had an impact on life satisfaction for the person with stroke and their caregiver [36]. Also, spousal stress has been associated with strokes in the left hemisphere, suggesting the effects of impairments in communicative competence [37]. A closer analysis of discordant dyads would be warranted in future qualitative and quantitative studies in order to broaden the knowledge regarding vulnerable dyads and their needs.

The results of the present study showed that 40% of the dyads were satisfied with life as a whole, representing a greater percentage of satisfied dyads compared with the two previous studies pertaining to combined life satisfaction who found 29% [20] and 30% satisfied couples [19]. One reason for this difference might be that the two previous studies had younger participants (median age 53 and 60 years) compared with the median age of 71 for the persons with stroke in the present study. Everyday life may be more demanding in working ages with other responsibilities compared with everyday life for a person that is retired [38]. Another reason for this difference might be that the previous studies investigated couples, where one of the persons has had a stroke. The group of caregivers that were not spouses or partners was relatively small in the present study, and may or may not have influenced the dyads combined life satisfaction. It is interesting to note however, that the dyads with combined low life satisfaction were partners indicating that being a partner might influence global life satisfaction to a greater extent than being a child or friend to the person with stroke.

The combination of two persons' life satisfaction into one unit establishes a certain relationship between the two individuals in the dyad, and this has been discussed in the literature. Bookwala and Schulz (1996) found that the well-being of one spouse was significantly associated with the well-being of the other in older adults living in the community [39]. Moreover, perceived needs of the caregivers may be inseparable from the needs of the care recipients [40]. Despite this, caution is advised in combining two individuals life satisfaction as was done in the present study. Studies show that caregivers and their recipients do not always agree upon the problems [23,41]. Despite the measuring of combined life satisfaction, it is also important to capture the perceptions of each person in the dyad and not risk losing the individuals' perspective. Qualitative longitudinal studies of dyads where one of the persons has had a stroke may help compare and contrast issues of individuality as well as mutuality in everyday life.

There were significant differences between the satisfied and the dissatisfied dyads in all SIS domains. A previous study on dyads with combined life satisfaction has shown that the satisfied groups perceived their participation in leisure activities and social life significantly greater than the dissatisfied group [20]. The present study supports this and provides more comprehensive information on the impact of stroke including all domains of the SIS, even physical and cognitive functioning as well as participation, in relation to combined life satisfaction. It is also interesting to note the differences in the SIS scores of the persons with stroke in the discordant dyads compared with either the satisfied or the dissatisfied dyads (see Table 4). The persons with stroke in the one group of discordant dyads experienced greater impacts in cognitive functions (i.e. memory and communication) while the other group had a tendency to experience predominantly physical impacts of their stroke. This is in line with other studies reported in a review article, showing that partners experience greater impact in quality of life when there are cognitive impairments involved compared to physical impairments [42].

The greatest impact of stroke was found in the domain participation regarding the persons with stroke in the dissatisfied dyads (see Table 4). A dyadic perspective regarding participation is thus warranted in future studies to determine if and how caregivers' participation is also affected.

### Limitations of the study

The caregivers' social relation to the individuals with stroke was not homogeneous in the present study, which might have affected the perceived life satisfaction [18]. Due to the relatively small sample size of those who were not partners, we chose not to further analyze dyads' life satisfaction with regards to social relations. There is a need for future studies with larger populations in order to identify if there are differences between caregivers who are partners or have other social relations to the person with stroke. However, in order to reflect reality as much as possible, all persons identified as a caregiver by the person with stroke were included in this study.

One strength of the present study was that it was hospital based and included even persons with aphasia. Persons with aphasia have been frequently excluded from stroke research according to a systematic review [43].

The usage of the division of LiSat-11 into satisfied and not satisfied can be questioned. However, this dichotomization has been found to be valid by the developers of the scale [18] and has been used in several other studies [20,26,27].

The small sample size limits the generalizability of the results. However, the study sample was extracted from a cohort who represented a population of all persons admitted to the stroke units during the period of one year. There were differences in the participants in the present study regarding age, gender and stroke severity compared with the persons making up the larger study, which could also limit the generalizability of the results. The larger study group was followed from stroke onset which is considered an advantage in comparison to a number of studies reported in a review article by White [42] showing that persons recruited to studies regarding caregivers were included when the patient was already receiving services and community support with the risk of misrepresenting the target population. Another limitation is that persons with mild stroke symptoms that may not have been admitted to the hospital or stroke unit or conversely, persons with such a massive stroke admitted to intensive care are underrepresented. The attrition rate was high for both persons with stroke and their caregivers which further calls for caution when extrapolating the results to other populations. There was missing data regarding the caregivers due to incomplete questionnaires returned by mail and possibly restricting the interpretation of the results.

### Clinical implications

Clinical implications regarding the dyad viewed as a single client could be incorporated in supportive interventions. The fact that the efficacy of interventions for caregivers of persons with stroke has not been confirmed [44] together with the predominant focus on the person with stroke in clinical guidelines within rehabilitation [41] motivates the need for a new perspective. A dyadic approach in supportive interventions and rehabilitation programs could be developed and evaluated.

Another clinical implication might be the implementation of client-centred rehabilitation interventions in the home setting. Home rehabilitation provides valuable contextual information [45] and fosters client and therapist partnerships [46] and may be especially conducive to individualized interventions based on the dyads' unique needs.

Knowledge regarding combined life satisfaction and the relationship to the perceived problems in everyday life could help expand our understanding of the dyads complex situation after stroke and facilitate identifying those persons in need of support. A greater understanding may than lead to effective rehabilitation interventions which would enable meaningful activities in everyday life and thereby affect life satisfaction for both individuals in the dyad.

## Competing interests

The authors declare that they have no competing interests.

## Authors' contributions

AB contributed to the design of the study, was responsible for data analysis and writing the manuscript. GE contributed to the design of the study and participated in data analysis, drafting and revision of the manuscript. LvK was responsible for initiating and planning the study as well as data collection and contributed to the design of the study, data analysis and to the revision of the manuscript. KT was responsible for initiating and planning the study, contributed to the design of the study and was instrumental in revision of the manuscript. All authors read and approved the final manuscript.
